# The genomic basis and environmental correlates of local adaptation in the Atlantic horse mackerel (*Trachurus trachurus*)

**DOI:** 10.1111/eva.13559

**Published:** 2023-06-06

**Authors:** Angela P. Fuentes‐Pardo, Edward D. Farrell, Mats E. Pettersson, C. Grace Sprehn, Leif Andersson

**Affiliations:** ^1^ Department of Medical Biochemistry and Microbiology Uppsala University Uppsala Sweden; ^2^ EDF Scientific Limited Cork Ireland; ^3^ Killybegs Fishermen's Organisation Donegal Ireland; ^4^ Department of Veterinary Integrative Biosciences Texas A&M University College Station Texas USA

**Keywords:** adaptation, conservation, genomics, management, marine fish, whole‐genome resequencing

## Abstract

Understanding how populations adapt to their environment is increasingly important to prevent biodiversity loss due to overexploitation and climate change. Here we studied the population structure and genetic basis of local adaptation of Atlantic horse mackerel, a commercially and ecologically important marine fish that has one of the widest distributions in the eastern Atlantic. We analyzed whole‐genome sequencing and environmental data of samples collected from the North Sea to North Africa and the western Mediterranean Sea. Our genomic approach indicated low population structure with a major split between the Mediterranean Sea and the Atlantic Ocean and between locations north and south of mid‐Portugal. Populations from the North Sea are the most genetically distinct in the Atlantic. We discovered that most population structure patterns are driven by a few highly differentiated putatively adaptive loci. Seven loci discriminate the North Sea, two the Mediterranean Sea, and a large putative inversion (9.9 Mb) on chromosome 21 underlines the north–south divide and distinguishes North Africa. A genome–environment association analysis indicates that mean seawater temperature and temperature range, or factors correlated to them, are likely the main environmental drivers of local adaptation. Our genomic data broadly support the current stock divisions, but highlight areas of potential mixing, which require further investigation. Moreover, we demonstrate that as few as 17 highly informative SNPs can genetically discriminate the North Sea and North African samples from neighboring populations. Our study highlights the importance of both, life history and climate‐related selective pressures in shaping population structure patterns in marine fish. It also supports that chromosomal rearrangements play a key role in local adaptation with gene flow. This study provides the basis for more accurate delineation of the horse mackerel stocks and paves the way for improving stock assessments.

## 1 INTRODUCTION

The extent to which marine species show genetic differentiation and local adaptation when no evident barriers restrict gene flow is a question of considerable interest in evolutionary biology, conservation, and management (Palumbi, 1994). Several marine species exhibit large population sizes, high gene flow, and minute genetic drift, resulting in low genetic differentiation that has been difficult to resolve with neutral genetic markers (Hauser & Carvalho, 2008).

Owing to advances in high‐throughput sequencing, recent genomic studies screening thousands to millions of genetic markers across the genome have revealed population structure and selection signatures in species previously assumed to be panmictic (e.g., Atlantic herring; Han et al., 2020) or lowly structured (e.g., Atlantic cod; Barth et al., 2017), Atlantic halibut (Kess et al., 2021). Population structure in marine fish has been characterized by shifts in allele frequencies at many small effect loci or fewer large effect loci (Gagnaire & Gaggiotti, 2016) and in chromosomal rearrangements (Akopyan et al., 2022; Han et al., 2020; Matschiner et al., 2022). Moreover, genomic divergence has been linked to ecological diversity, for example, in migratory behavior (Kirubakaran et al., 2016), seasonal reproduction (Lamichhaney et al., 2017), or along environmental gradients (Han et al., 2020; Stanley et al., 2018). Therefore, a thorough examination of genomic variation, including neutral and adaptive loci, can help identify distinct biological units and genetic variants associated with local adaptation. This is knowledge of great interest in conservation and management, especially in the face of climate change.

Fish stock identification is an important prerequisite for fisheries assessment and management (Cadrin & Secor, 2009), however, many exploited stocks have traditionally been defined according to geographical and political features rather than on a biological basis. Such is the case in the European Union, where the term “stock” is defined as “a marine biological resource that occurs in a given management area.” (Anon, 2014) As more information becomes available, it is evident that the temporal and spatial distributions of most fisheries resources are not aligned to these artificial divisions (Kerr et al., 2017) and that biological populations are more dynamic and complex (Reiss et al., 2009; Stephenson, 2002). Therefore, it is critical to identify the underlying population structure and use this information to identify the appropriate level at which to define assessment and management units. It is also important to be able to assign individuals in mixed surveys and commercial catches to the population or assessment unit to which they belong in order to obtain accurate estimates of population size and fishing pressures to which they are exposed (Casey et al., 2016; Hintzen et al., 2015).

The Atlantic horse mackerel (*Trachurus trachurus* Linnaeus, 1758) is a marine benthopelagic shoaling fish widely distributed in the east Atlantic, from Norway to southern Africa (FAO Major Fishing areas 27, 34 and 47) and in the Mediterranean Sea (FAO Major Fishing area 37) (Froese & Pauly, 2021). Its extensive range implies that populations may be exposed to diverse environmental conditions (e.g., temperature, salinity, oxygen, turbidity, mineral content; Liu & Tanhua, 2021; Schroeder et al., 2016; Shi & Wang, 2010) and selective pressures, making this species ideal for the study of local adaptation. Horse mackerel are generally found in continental shelf waters (100–200 m depth) but are also present in deeper (~1000 m) or near‐shore waters. The species undertakes annual migrations between spawning, feeding, and over‐wintering areas (Abaunza et al., 2003), though these are not well documented and the interaction between adjacent stocks or populations is not clear. Horse mackerel is considered to be an asynchronous batch spawner with indeterminate fecundity and it is unknown if they are faithful to their original spawning grounds (Gordo et al., 2008; Ndjaula et al., 2009). Eggs and larvae are pelagic and are typically either found over the continental shelf, from the surface to 100 m depth, or near the coast (Alvarez & Chifflet, 2012; van Beveren et al., 2016).

In the northeast Atlantic, horse mackerel are assessed and managed as three main stocks: the Western, the North Sea, and the Southern stocks (Figure S1), which were largely defined based on the results of the HOMSIR project (Abaunza et al., 2008). Populations inhabiting coastal waters along Morocco and Mauritania, in northwest Africa, are considered a separate group, denominated the “Saharo‐Mauritanian stock.” However, the populations belonging to this stock are less studied and monitored than those in the north. The age and length at 50% maturity are estimated to be 3–4 years and 23–24 cm for the Western stock (ICES, 2022b) and 2–3 years and 19–21 cm for the Southern stock (ICES, 2022a). There is no information available about the age or length‐at‐maturity of the North Sea stock (ICES, 2022b). The discreteness of the three main stocks, as well as the location and levels of mixing between them, is unknown, which leads to uncertainty in the input data for stock assessments. Previous genetic studies on Atlantic and Mediterranean horse mackerel using traditional methods such as mitochondrial DNA and microsatellite markers indicated low genetic differentiation and provided inconclusive results in regard to population substructuring beyond the three main stocks (Brunel et al., 2016; Cimmaruta et al., 2008; Comesaña et al., 2008; Farrell & Carlsson, 2018; Healey et al., 2020; Kasapidis & Magoulas, 2008; Mariani, 2012; Sala Bozano et al., 2015).

Given the elusive nature of the population structure of the Atlantic horse mackerel and its ecological and commercial importance in the east Atlantic, we asked whether the current stock divisions reflect biological groups defined by genetics and whether the environment drives patterns of population subdivision and local adaptation. Therefore, the aims of this study were to (i) identify the population structure underlying the stock divisions; (ii) estimate the extent of genetic differentiation between populations based on whole‐genome sequencing; (iii) identify the evolutionary processes, genetic basis, and environmental drivers of local adaptation; and (iv) design a genetic tool (SNP panel) that can be used for future population studies and genetic stock identification.

## 2 MATERIALS AND METHODS

### 2.1 Sampling and DNA isolation

**FIGURE 1:**
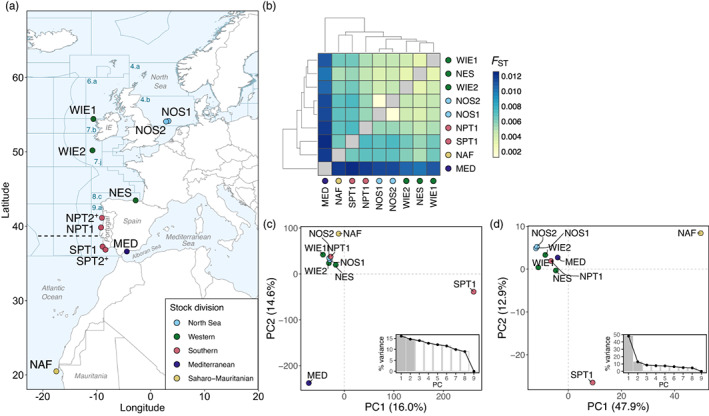
Sampling sites and population structure of the Atlantic horse mackerel. (a) Map depicting the 11 sampling sites in the east Atlantic Ocean. ICES fishing divisions are denoted with dark blue lines, the same as their alphanumerical code. The approximate location of a biogeographical transition zone in central Portugal, near Lisbon, is denoted with a horizontal dashed line (Cunha, 2001; Santos et al., 2007). In all plots, each dot represents a sampling location and its color indicates the corresponding ICES stock division (ICES, 2005) after the HOMSIR project (Abaunza et al., 2008). (b) Heatmap plot representing pairwise pool‐*F*_ST_ values based on ~12.8 million SNPs. Actual values are available in Figure S9A. (c, d) Principal component analysis (PCA) plot based on (c) undifferentiated (61,543 SNPs) and (d) highly differentiated (818 SNPs) markers. The first two axes are shown. Inset bar plots in each PCA plot show the percentage (%) of genetic variance explained by the first nine principal components (PC). Note that the samples NPT2 and SPT2 (marked with a + sign), which are spatial replicates of NPT1 and SPT1, were excluded from analyses (b) to (d), as technical artifacts could not be excluded in a pilot analysis. Sample names are abbreviated as in Table 1.

Samples were collected opportunistically between 2015 and 2017 through existing fishery surveys, fisheries targeted to horse mackerel, and as bycatch at 11 locations across the eastern Atlantic and the western Mediterranean Sea (Figure 1, Table 1, and Table S1). Maturity stages were recorded by sample collectors using different maturity keys. Therefore, these were standardized to the 6‐point international horse mackerel maturity scale (Table S2; ICES, 2015). We aimed to collect spawning fish to ensure that samples could provide a valid baseline. However, due to the opportunistic nature of sampling, this was not always possible (Table S3). The North Sea samples comprised primarily juvenile stage 2 maturing fish in 2016 (NOS1) and mature spawning fish in 2017 (NOS2). The samples from the Western stock area, west of Ireland (WIE1–WIE2), and the Northern Spanish Shelf (NES), were primarily at spawning conditions. The Portuguese samples contained a mix of length classes and maturity stages, with primarily juvenile samples in 2016 (NPT1, SPT1) and adult samples in 2017 (NPT2, SPT2). Very few spawning individuals were available from Portuguese waters. The North African samples were primarily spent individuals and no biological information was available for the Mediterranean samples. A 0.5 cm^3^ piece of tissue was excised from the dorsal musculature of each specimen and stored at 4°C in absolute ethanol. Total genomic DNA was extracted from the majority of samples by Weatherbys Scientific Ltd, Ireland from 30 mg of tissue using sbeadex™ magnetic bead‐based extraction chemistry on the LGC Oktopure™ platform. Samples from the Mediterranean Sea were extracted using CTAB, and some samples from Portugal (NPT2, SPT2) were extracted with Chelex and proteinase‐K based extraction protocol (Table S1). The Chelex protocol produced single‐stranded DNA, whereas the other methods, double‐stranded molecules. DNA quantity was measured with a NanoDrop ND‐1000 spectrophotometer.

**TABLE 1:** Collection details of the 11 Atlantic horse mackerel samples included in this study.

Pool ID	Stock^a^	Area	Year	Latitude	Longitude	Sample size
WIE1	Western	West of Ireland	2016	54.42	−10.62	51
WIE2	Western	West of Ireland	2017	52.76	−11.00	62
NOS1	North Sea	Southern North Sea	2016	54.15	3.30	96
NOS2	North Sea	Southern North Sea	2017	54.07	2.85	70
NPT1	Southern	Northern Portugal	2016	39.83	−9.20	64
SPT1	Southern	Southern Portugal	2016	37.26	−8.92	30
NPT2	Southern	Northern Portugal	2017	41.14	−9.03	47
SPT2	Southern	Southern Portugal	2017	36.84	−8.38	48
NAF	Saharo‐Mauritanian	North Africa	2016	20.20	−17.50	57
NES	Western	Northern Spanish shelf	2016	43.59	−2.78	96
MED	Mediterranean	Alboran Sea	2018	36.49	−4.42	49

^a^ Stock divisions from ref. Abaunza et al. (2008).

### 2.2 Pool library preparation and sequencing

We generated pooled DNA whole‐genome sequencing (pool‐seq) data to assess the genomic variation among samples. This method provides population‐level allele frequencies by sequencing to a high depth a single‐barcoded library prepared from a mixture (pool) of DNA of individuals from a population (Schlötterer et al., 2014), which implies individual information is lost. DNA pools were prepared by mixing equal amounts of DNA of 30–96 individuals collected in close spatial and temporal proximity (Table S1). DNA pools were quantified using a Qubit Fluorometer (Thermo Fischer Scientific Inc.) aiming to have at least 1.5 μg of DNA in 25–50 μL and were submitted to the SNP&SEQ Technology Platform in Uppsala, Sweden, for library preparation and high‐throughput sequencing. A PCR‐free Illumina TruSeq library with an insert size of 350 base pairs (bp) was prepared for most pools, except for the ones extracted with Chelex (NPT2, SPT2), for which a Splinted Ligation Adapter Tagging (SPLAT) library was used instead, as it is aimed for single‐stranded DNA (Raine et al., 2017). Paired‐end short reads (2 × 150 bp) were generated using an Illumina NovaSeq sequencer and S4 flow cells.

### 2.3 Read mapping and variant calling

Low‐quality bases, sequencing adapters, and reads with length <36 bp were removed from the raw read data set using *Trimmomatic* v.0.36 (Bolger et al., 2014). This yielded 490–764 million high‐quality reads per pool. Clean reads were mapped against the *Trachurus trachurus* genome assembly (Accession: GCA_905171665.1; Genner & Collins, 2022) using *bwa‐mem* 0.7.17 (Li, 2013), and ~98%–99% of the reads aligned with high mapping quality to the genome assembly.

Variant calling was performed using the algorithm *UnifiedGenotyper* of *GATK* v3.8 (McKenna et al., 2010). Biallelic SNPs were retained and various quality filters were applied to remove spurious markers (e.g., based on GATK variant quality scores, Figure S2, depth of coverage (DP) per sample, Figure S3, and others; more details can be found as extended Materials and Methods in the Appendix S1). The resulting high‐quality SNPs were used in further analyses. A summary of the data generation steps is shown in Figure S4.

### 2.4 Population genetic structure and genetic diversity

We assessed population structure with pairwise pool‐*F*_ST_ and principal components analysis (PCA). For all population pairs, we calculated pool‐*F*_ST_ (${\hat{F}}_{\mathrm{ST}}^{\text{pool}}$) and its 95% confidence interval (CI) using the R package *poolfstat* (Hivert et al., 2018). This pairwise pool‐*F*_ST_ statistic is equivalent to Weir & Cockerham's *F*_ST_ (Weir & Cockerham, 1984) and accounts for random chromosome sampling in pool‐seq. The 95% CI was calculated based on a block‐jackknife sampling estimation of *F*_ST_ standard error over blocks of 1000 consecutive SNPs. Statistical significance of the difference in mean‐*F*_ST_ between genetically distinct groups was assessed with a Wilcoxon test and an alpha significance level of 0.001. A pilot analysis indicated that two pool samples from northern and southern Portugal (NPT2, SPT2) had an unusual depth of coverage and insert size distributions (Figure S5A,B) and behaved as outliers in a PCA (Figure S5C). Since the DNA extraction and sequencing library methods applied to these samples were different from those used in the rest of the samples, it could not be ruled out that their unusual behavior was due to technical artifacts. Therefore, these samples were omitted from all analyses.

To evaluate whether neutral or selective processes better explain the observed patterns of genome‐wide differentiation, we separately performed PCA on two SNP subsets, one of undifferentiated (i.e., presumably neutral) and the other of highly differentiated markers (outliers, assumed to have been subject to selection). Both marker sets were chosen based on the empirical distribution of allele frequencies and standard deviation (SD) cutoff values (Figure S6; see Appendix S1 for details). To reduce redundancy and physical linkage among SNPs, in the undifferentiated marker set, we retained one SNP every 1 kb, and in the differentiated marker set, one SNP every 10 kb, as the linkage is expected to be more pronounced in regions under selection. PCA was separately performed on each market set using the *R* package *prcomp*.

To examine the genome‐wide variation of genetic diversity in each pool, we calculated nucleotide diversity (*π*) per pool in 10 kb‐sliding windows with a step size of 2 kb using *PoPoolation 1.2.2* (Kofler et al., 2011; see Appendix S1 for details). Plotting and statistical testing were performed using the *R* environment (R Core Development Team, 2023).

Furthermore, we evaluated whether population structure resulted from spatially limited gene flow (isolation‐by‐distance, IBD) by conducting a linear regression of the linearized genetic distances (linearized‐${\hat{F}}_{\mathrm{ST}}^{\text{pool}}$ = $\frac{{\hat{F}}_{\mathrm{ST}}^{\text{pool}}\;}{1-{\hat{F}}_{\mathrm{ST}}^{\text{pool}}}$; Rousset, 1997) to the geographical distance between locations. Geographical distances were calculated as the straight‐line distance in kilometers (km) (“as the crow flies”) with the *R* package *geosphere* (Hijmans, 2017). We examined IBD for all samples and separately for the northern samples only, while excluding the replicate sample from the North Sea (NOS2) as it potentially represents the same cohort as NOS1 and therefore it does not serve as a spatial replicate. The statistical significance of IBD was evaluated with a Mantel test and 1000 permutations using the *R* package *ade4* (Dray & Dufour, 2007).

### 2.5 Detection of loci under selection

We applied effective coverage correction (*n*_eff_) to the raw read counts, in order to account for the random variation of read coverage and chromosome sampling during pooling and sequencing (Bergland et al., 2014; Feder et al., 2012; Kolaczkowski et al., 2011; see Appendix S1 for details). The corrected read counts were then used to calculate pool allele frequencies. Custom scripts developed for these calculations are publicly available in the repository referenced in the Data Archiving Statement.

To identify genomic regions with elevated differentiation with respect to the genomic background that was characteristic of particular populations, we calculated the absolute delta allele frequency (dAF) per SNP between paired contrasts of grouped pools, as dAF = absolute (meanAF (group1) – meanAF (group2)). The contrasts evaluated were established based on geographic closeness, PCA clustering, and biological knowledge (Table S4). To identify regions with consistent differentiation across nearby SNPs, we also calculated the moving average of dAF in windows of 100 consecutive SNPs. This statistic helps to smooth out the signal and is not dependent on single outlier SNPs caused by random effects of pool‐seq. With a heatmap plot, we further explored the concordance in allele frequencies among samples of the most differentiated SNPs per locus and contrast. Only in this case, we included the two samples from Portugal that were presumably affected by technical bias (NPT2, SPT2), as they showed similar allele frequencies at outlier loci as the other Portuguese samples (NPT1 and SPT1). All analyses were performed using *R* and plotting was done with the package *ggplot2* (Wickham, 2016).

### 2.6 Validation of informative markers for genetic stock identification

To validate pool‐seq findings and to identify a panel of highly informative SNPs for genetic stock identification, we obtained the genotypes of 160 individuals (20 fish each from eight locations, Table S5) in 100 SNPs. The 100‐SNPs panel consisted of 24 neutral markers and 76 putatively adaptive markers (see Appendix S1 for details; Figure S7). The split of adaptive markers, in terms of observed association, was North Sea (*n* = 28), the 9.9 Mb putative inversion underlying the north–south genetic pattern (*n* = 12), west of Ireland (*n* = 14), Alboran Sea (*n* = 13), southern Portugal (*n* = 4), north Africa (*n* = 4). Three to four individuals per location were genotyped twice to assess genotyping error rate. DNA extraction and SNP genotyping were undertaken by IdentiGEN, Ireland, using their IdentiSNP genotyping assay chemistry. The protocol utilizes target‐specific primers and universal hydrolysis probes. Following an end‐point PCR reaction, different genotypes are detected using the Araya fluorescence reader (LGC Biosearch Technologies, UK).

Based on individual allele frequencies, we undertook a preliminary analysis of population structure among the eight genotyped fish aggregations. It should be noted that sample sizes were small and therefore the results of the population analyses should be viewed as preliminary until further large‐scale screening is undertaken. Only individuals and markers with >80% genotyping success were retained. Deviations from Hardy–Weinberg equilibrium (HWE) and linkage disequilibrium (LD) were assessed with *Genepop* 4.2 (Rousset, 2008). *Microsatellite Analyzer* (*MSA*) 4.05 was used to calculate pairwise *F*_ST_ estimates (Dieringer & Schlötterer, 2003). In all cases with multiple tests, significance levels were adjusted using the sequential Bonferroni technique (Rice, 1989). PCA was performed using the *R* function *prcomp*.

We estimated admixture coefficients, which represent the proportion of an individual genome that originates from multiple ancestral gene pools (or ancestral source populations, *K*), using the *sNMF* algorithm (Frichot et al., 2014) of the *R* package *LEA* (Frichot et al., 2015). We tested *K* = 1–9, with 10 repetitions and 200 iterations. The most likely *K* corresponds to the value where the cross‐entropy criterion (metric that evaluates the error of the ancestry prediction) plateaus or increases (Frichot et al., 2014). We plotted the average admixture proportions per population sample over a map using the *R* packages *ggplot* (Wickham, 2016) and *ggOceanMaps* (Vihtakari, 2020).

### 2.7 Characterization of a putative inversion on chromosome 21

To assess and compare the genetic diversity and spatial distribution of haplotypes of the putative inversion on chromosome (chr) 21, we extracted the individual genotypes of 12 diagnostic SNPs within the inversion from the 100‐SNP data set (Figure S7). We performed a PCA with the *R* function *prcomp* to identify the genotype of each individual. Individuals were assigned to a haplotype group using the first two eigenvectors of the PCA and the *k*‐means clustering algorithm implemented in the *R* function *kmeans*. We calculated observed heterozygosity for each of the PCA clusters, with the expectation that the middle cluster, presumably corresponding to inversion‐level heterozygotes, will have the highest heterozygosity. These analyses and correspondent graphics were performed using *R*.

### 2.8 Genome–environment association

To identify which environmental variables are related to adaptive genetic variation and local adaptation, we evaluated genome–environment associations (GEA) with a redundancy analysis (RDA) implemented in the *R* package *vegan* (Dixon, 2003). RDA is a constrained ordination method that allows modeling of linear relationships of multiple response variables (genetic variation) on multiple explanatory variables (environment predictors). Thus, in landscape genomics applications, this method allows the identification of allele frequencies that covary with environmental variables (Capblancq & Forester, 2021). We retrieved data layers of eight environmental parameters from *Bio‐Oracle* v.2.1 (Assis et al., 2018; Tyberghein et al., 2012) and extracted values for each sampled location using the *R* package *sdmpredictors* (Bosch, 2020; Table S8). The environmental parameters corresponded to mean depth seawater temperature (°C), *Tmean*; temperature range (*Trange*); nitrate concentration (μmol/m), *NO*_3_; iron concentration (μmol/m^3^), *Fe*; current velocity (m/s), *CVel*; primary production (g/m^3^/day); seawater salinity (PSS); and dissolved oxygen concentration (μmol/m^3^). Prior to RDA, environmental data were standardized to zero mean and unit variance and some of the highly correlated variables were removed (|*R* ^2^ ≥ 0.7|) (see Appendix S1 for details, Figure S8). To perform an adaptively enriched RDA, we used the uncorrelated and statistically significant environmental parameters and the pool‐allele frequencies of the 10 most differentiated SNPs in each divergent genomic region identified with genome scans (*N* = 136). The sample NOS2 was excluded from this analysis as it is potentially a temporal replicate of NOS1, and thus, it cannot serve as a spatial replicate. The statistical significance of the RDA model, constrained axes, and environmental variables was assessed with 1000 permutations. Candidate SNPs corresponded to those with the highest loadings on significantly constrained axes (>1 standard deviation, SD, of the loadings' distribution). Based on the coefficient of determination (*R* ^2^), we identified which of the environmental variables each candidate SNP is most strongly correlated with. We further explored the linear relationship between candidate SNPs and environmental predictors using a scatterplot, and the genetic patterns between samples with a heatmap plot depicting allele frequencies of candidate SNPs.

### 2.9 Functional annotation of gene models

The gene models of the Atlantic horse mackerel genome were developed by Ensembl (2021) and became available in an Ensembl Rapid release in March 2021 (Howe et al., 2021). However, these gene models were lacking gene symbols (names) and descriptions (only gene IDs were available). Given that these annotations are relevant for the interpretation of results, we ran the functional gene annotation pipeline developed by the National Bioinformatics Infrastructure Sweden (NBIS, Binzer‐Panchal et al., 2021) to retrieve this information (see Appendix S1 for details). Additionally, for the top 2% most differentiated SNPs within each divergent genomic region detected with genome scans, we annotated the closest overlapping gene (up to ±40 kb) and the variant effect prediction (e.g., missense, synonymous, upstream, downstream, intergenic) using *snpEff* v.4.1 (Cingolani et al., 2012).

## 3 RESULTS

### 3.1 Population genetic structure

We generated pooled DNA whole‐genome sequence data of 11 Atlantic horse mackerel samples across the species' range in the east Atlantic Ocean and the western Mediterranean Sea (Figure 1a, Table 1). We aimed to sample reproductive units that could be representative of baselines, however, only 37% of collected fish were in spawning condition (Table S3, maturity categories described in Table S2). Therefore, it is possible that the levels of population structure here described are underestimated, as that individual information is lost in pool‐seq data and, with it, the possibility to identify migrants. Each pool had a mean depth of coverage between 25.7× and 46.3× (Table S6). After variant calling with *GATK*, a total of ~12.8 million biallelic SNPs passed quality filters and were used in the population analysis.

The pairwise pool‐*F*_ST_ estimates (Figure 1b, Figure S9a) and their 95% confidence intervals (Figure S9b) indicated low genome‐wide differentiation among samples (global mean pool‐*F*_ST_ = 0.007 ± 4.4e‐05) but revealed three subtle population structure patterns that were statistically significant (adjusted *p*‐values of the Wilcoxon test *p*adj_wilcox_, <0.0001, Figure S9b). First, the largest genomic differences exist between the western Mediterranean Sea and all Atlantic samples (mean pool‐*F*_ST_ = 0.010–0.012, Figure S9a, comparison respect northern samples and Mediterranean *p*adj_wilcox_ = 5.4e‐06, respect southern samples and Mediterranean *p*adj_wilcox_ = 2.6e‐05, Figure S9b). Second, Atlantic samples genetically differ following a latitudinal pattern with a break near mid‐Portugal (mean pool‐*F*_ST_ = 0.008, Figure S9a, respect northern and southern samples *p*adj_wilcox_ = 4.6e‐07), where samples north of this break (“northern” samples: North Sea—NOS1, NOS2, west of Ireland—WIE1, WIE2, north of the Spanish Shelf—NES, and north of Portugal—NPT1) are genetically more similar to each other (mean pool‐*F*_ST_ = 0.004, Figure S9a) than samples south of this break (“southern” samples: south of Portugal—SPT1 and north of Africa—NAF) (mean pool‐*F*_ST_ = 0.006, Figure S9a, Figure 1b). Third, the two samples from the North Sea (NOS1 and NOS2) show the highest genetic similarity (pool‐*F*_ST_ = 0.001, Figure S9a) of all and are distinguished from other “northern” samples (mean pool‐*F*_ST_ = 0.0045, Figure S9a,b).

A pattern of isolation‐by‐distance was not detected when considering all samples (linear regression Figure S10a, Mantel *r* = 0.057, *p* = 0.29) or only the northern ones (linear regression Figure S10b, Mantel *r* = 0.43, *p* = 0.12), implying that the observed patterns of population structure are not derived from spatially limited gene flow.

We further examined whether population structure patterns were driven primarily by neutral or selective processes by separately performing PCA for two SNP subsets, of undifferentiated (neutral) or highly differentiated (under selection) markers. These marker sets were chosen based on the standard deviation in allele frequencies across populations (Figure S6, see the Appendix S1 for details). In the PCA based on neutral markers (61,543 SNPs, Figure 1c) the first two axes explained 30.6% of the genetic variance. The samples from southern Portugal (SPT1) and the Mediterranean (MED) were clearly separated from others, while the North Africa (NAF) sample was genetically closer to the northern samples. The North Sea samples tightly are clustered with other northern samples, being almost indistinguishable. In contrast, in the PCA with selective makers (818 SNPs, Figure 1d), the first two axes explained twice as much genetic variance (60.8%). The samples from southern Portugal (SPT1) and North Africa (NAF) stood out. The Mediterranean Sea sample was genetically closer to the northern samples, and the two North Sea samples clustered closely together and were separated from other northern samples.

Taken together, these results indicate that the separation between the westernmost part of the Mediterranean Sea and Atlantic populations might be driven by neutral processes, while the latitudinal pattern and separation of North Sea samples could be the result of selective processes.

### 3.2 Putative loci under selection

**FIGURE 2:**
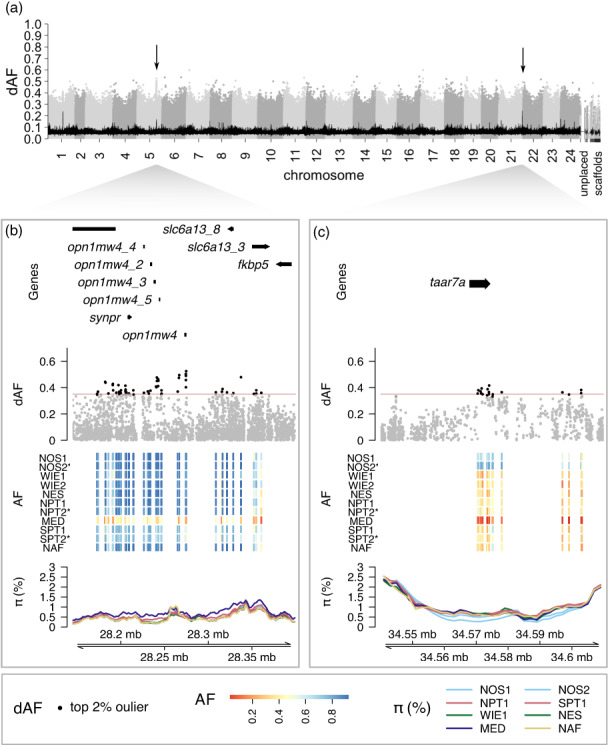
Two selective regions are distinctive of the western Mediterranean Sea. (a) Manhattan plot depicting the dAF per SNP across the genome for the contrast between the Alboran Sea and all other samples. Each dot is a single SNP, and alternating gray tones were used to differentiate SNPs in consecutive chromosomes. The black line is the rolling average of dAF over 100 SNPs. Regions of interest are indicated with an arrow. (b, c) Zoom‐in plots for the signals on (b) chr 5 and (c) chr 21. Each zoom‐in plot consists of four tracks: the first, a representation of the gene models; the second, the dAF of SNPs, with the top 2% most differentiated SNPs denoted in black; the third, a heatmap plot representing the pool minor allele frequency per sample (rows) of the top 2% SNPs (columns), in which temporal replicates are denoted with an asterisk; and the fourth, the percentage of nucleotide diversity (*π*) calculated in 10 kb windows with 2 kb step size along the region with a separate line for each sample, colored based on the ICES stock divisions. Sample names are abbreviated as in Table 1.

**FIGURE 3:**
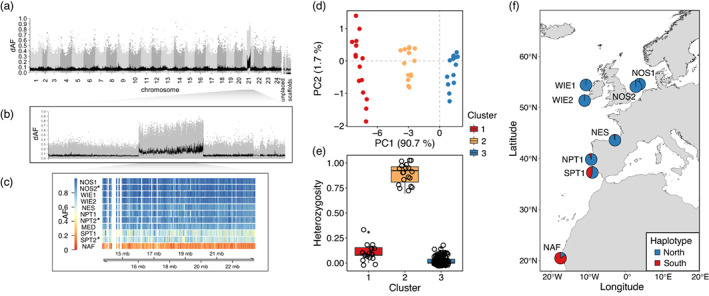
Putative chromosomal inversion on chromosome 21 underlies a latitudinal genetic cline. (a) Manhattan plot representing the absolute difference in pool‐allele frequencies (dAF) of each SNP along the genome for the contrast between North Africa versus other populations (see Section 2 for details). The *x*‐axis shows the genomic position of each SNP and the *y*‐axis, its dAF value. SNPs in consecutive chromosomes are distinguished with alternating gray tones. The black line toward the bottom of the plot corresponds to the 100 SNPs‐rolling average of dAF values. (b) Close‐up Manhattan plot to chromosome 21. (c) Pool‐allele frequency per sample (rows) of the top 2% markers (columns) within the putative inversion, temporal replicates are denoted with an asterisk. (d–f) Analysis of inversion frequency based on the genotype of 15 SNPs screened in 160 individuals. (d) PCA plot showing individual clustering with respect to the inversion genotype. (e) Individuals observed heterozygosity in each PCA cluster. Clusters “1” and “3” correspond to homozygous “southern” and “northern” individuals, respectively, whereas cluster “2” is heterozygous individuals. (f) Map showing the geographic distribution of inversion haplotypes across sampled locations. Sample names are abbreviated as in Table 1.

**FIGURE 4:**
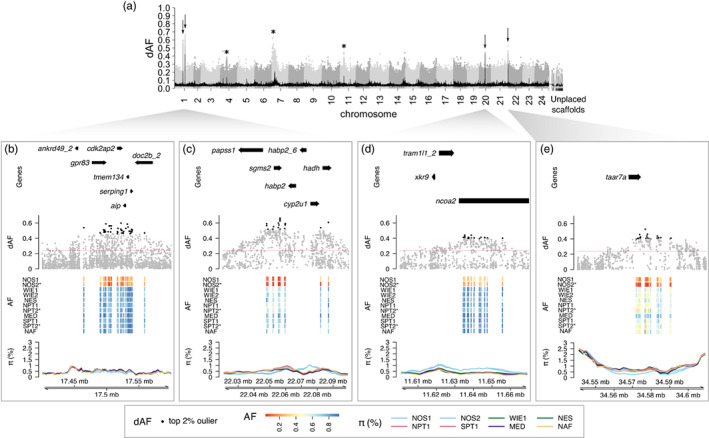
Genomic regions characteristic of the North Sea. (a) Manhattan plot representing the dAF of each SNP along the genome for the contrast between the North Sea and all other samples. Each dot is a single SNP, and alternating gray tones were used to differentiate SNPs in consecutive chromosomes. The black line toward the bottom is the rolling mean of dAF over 100 SNPs. (b–e) Close‐up plots of the four most divergent regions (highlighted with arrows in (a)) on (b, c) chr 1, (d) chr 20, and (e) chr 21. Plots of the other differentiated regions on chr 4, 7, and 11 are shown in Figure S11 (highlighted with an asterisk in (a)). Each close‐up plot consists of four tracks (from top to bottom): The first, illustrates gene models; the second corresponds to the dAF of SNPs, in which the top 2% of markers are denoted in black. The horizontal red line indicates the Bonferroni *Z*‐score threshold of significance; the third track is a heatmap plot depicting the pool‐allele frequency per sample (rows) of the top 2% SNPs (columns), temporal replicates are denoted with an asterisk; the fourth track is the percentage of nucleotide diversity (*π*) for each sample calculated over 10 kb sliding windows with a step size of 2 kb. The color of each line indicates the designated ICES stock division of each pool. Sample name abbreviations as in Table 1.

We performed genome scans based on the absolute difference in allele frequencies (dAF) per SNP for paired contrasts to identify outlier loci (presumed under selection) that are characteristic of certain populations. The contrasts were chosen based on PCA clustering patterns (Figure 1c,d). Outlier genomic regions were identified using a Bonferroni *Z*‐score threshold of significance. This analysis revealed a number of genomic regions with elevated differentiation for three contrasts: (i) the Mediterranean Sea versus others, (ii) north Africa versus others, and (iii) the North Sea vs. others (Figures 2, 3, 4). A summary of the 2% most differentiated SNPs per region, their closest gene, gene function, and relative position to genes (e.g., missense, synonymous, upstream, downstream, intergenic) inferred with *snpEff* are compiled in Table S7.

#### 3.2.1 Two outlier loci distinguish the western Mediterranean from Atlantic samples

Besides genome‐wide differences, the contrast between the Mediterranean Sea and all other samples revealed two distinctive regions at chromosomes (chr) 5 and 21 (Figure 2). In this case, the signals of divergence were not as evident as in other contrasts given the overall higher genome‐wide differentiation of this sample. Thus, we focused on the peaks in the rolling average of dAF values (Figure 2a), as it requires consistent allele frequency differences for the contrast of interest. We considered candidate genes those overlapping or located in the vicinity of the 2% most differentiated SNPs in each locus. In the region on chr 5 (Figure 2b), the Mediterranean Sea sample had intermediate allele frequencies across loci and higher nucleotide diversity, while other samples tended to be fixed for one haplotype and showed lower nucleotide diversity. The main candidate gene in this region is *opn1mw4* (*green‐sensitive opsin‐4*). In the putative selection signal on chr 21 (Figure 2c), the Mediterranean sample had one haplotype close to fixation, the opposite haplotype was in high frequency in the North Sea, and intermediate frequencies were prevalent elsewhere. This region harbors a single gene, *taar7a* (*trace amine‐associated receptor 7A*). The current knowledge of known functions of candidate genes is presented in Table S7.

#### 3.2.2 A presumed 9.9 Mb inversion distinguishes populations along a latitudinal pattern

In the north Africa versus other samples contrast, we discovered that a single large region on chr 21 underpins the latitudinal genetic pattern distinguishing “northern” and “southern” samples with respect to a break along mid‐Portugal (Figure 3). This locus consists of a block of several SNPs with elevated dAF spanning 9.9 Mb (Figure 3a,b). The large size and abrupt drop in allele frequency differences towards the edges are characteristic of structural variants with suppressed recombination (e.g., inversions) that do not represent recent selective sweeps (Han et al., 2020). An analysis of the pool‐allele frequencies of the 2% most differentiated SNPs at this locus (Figure 3c) revealed striking allele frequency differences between the “northern” samples (including the Mediterranean Sea) and North Africa, while intermediate allele frequencies were prevalent in southern Portugal.

To further characterize this structural variant, we leveraged the genotype data of 160 individuals for 12 SNPs within this region. As expected for a chromosomal inversion, in a PCA plot individuals were separated into three clusters depending on their genotype (Figure 3d), and individuals in the intermediate cluster exhibited the highest mean heterozygosity (Figure 3e). Overall, the individual genotype data supported the observations made with pool‐seq data regarding the spatial distribution of inversion haplotypes, although it offered greater resolution. For instance, it revealed that the “northern” haplotype dominates in the North Sea, west of Ireland, northern Spanish shelf, and northern Portugal. The “southern” haplotype is predominant in north Africa, and common in southern Portugal (Figure 3f). This putative inversion contains about 1077 genes, making it difficult to infer which particular genes are under selection.

#### 3.2.3 A genetically distinct population in the southern North Sea

The contrast between the North Sea and all other samples revealed seven genomic regions with elevated differentiation on chr 1, 4, 7, 11, 20, and 21 (Figure 4a). Further examination of allele frequencies of the most differentiated SNPs at each locus indicated that the two North Sea samples collected 1 year apart (NOS1–NOS2) were very similar genetically and that their allele frequency patterns were distinctive of this location (Figure 4b–e, Figure S11). For instance, in the selection signals on chr 1 and 21, North Sea samples had variant alleles close to fixation and a slight reduction in nucleotide diversity (*π*; Figure 4b,c,e). At the outlier loci on chr 4, 11, and 20, North Sea samples had predominantly intermediate allele frequencies, with similar (chr 4 and 11, Figure S11b,d) or higher (chr 20, Figure 4d) nucleotide diversity with respect to other pools. Additional divergent regions on chr 7 and 11 showed a similar but noisier pattern, presumably due to the presence of complex structural variants at these loci (Figure S11c,d).

The most striking signals were located on chr 1, followed by those on chr 20 and 21 (Figure 4). The signal on chr 1 encompasses two regions (chr1:17.4–17.6 Mb, chr1:22.0–22.1 Mb). In the first region (Figure 4b), the most differentiated SNPs are in the vicinity of the gene *gpr83* (G‐protein coupled receptor 83). In the second region (Figure 4c), the top candidate gene is *sgms2* (*sphingomyelin synthase 2*) based on its location. The signals on chr 20 (Figure 4d) and chr 21 (Figure 4e) contain a single gene each, *ncoa2* (*nuclear receptor coactivator 2*) and *taar7a* (*trace amine‐associated receptor 7A*). Note that the locus on chr 21 is the same as the one described in the Mediterranean sample (Figure 2c), in which the North Sea samples and the Mediterranean samples tend to be fixed for different alleles. Additional selection signals on chr 4, 7, and 11 (Figure S11) were not as clear as the ones just described (e.g., smaller allele frequency differences and/or inconsistent allele patterns). Therefore, it was difficult to identify candidate genes in these regions. The functional annotation of positional candidate genes in differentiated genomic regions is summarized in Table S7.

#### 3.2.4 Short‐term stability of adaptive variants

Four geographic areas were sampled twice, 1 year apart, in the North Sea (NOS1–NOS2), western Ireland (WIE1–WIE2), northern Portugal (NPT1–NPT2), and southern Portugal (SPT1–SPT2). While the replicate samples from Portugal (NPT2, SPT2) were excluded from all analyses due to potential technical bias, for exploratory purposes, they were included in the heatmap plot representing allele frequencies of the most differentiated SNPs in putatively adaptive loci (Figures 2b,c, 3c and 4b–e). We observed high concordance in the genetic composition of adaptive loci between short‐term replicates and among northern and southern Portuguese samples. However, as the replicate samples were collected within a relatively short‐time period, it is possible that the same cohort was sampled twice. Examination of the length–frequency and maturity stages (Table S3) indicates that in the North Sea, the modal length of the 2016 samples was 21 cm and of the 2017 samples was 22 cm. Therefore, it is possible that the short‐term replicates comprised the same cohort. Regardless, the samples were collected 1 year apart and do at least support the presence of short‐term stability of the horse mackerel in this area at this time. The modal length of the 2017 west of Ireland sample was smaller than the 2016 samples indicating that these samples contained predominately different cohorts. The Portuguese samples were more likely to contain multiple cohorts (see ICES, 2022a) and also comprised a mix of northern and southern type horse mackerel and, as such, it is difficult to assess temporal stability in this area.

### 3.3 Genome–environment associations

**FIGURE 5:**
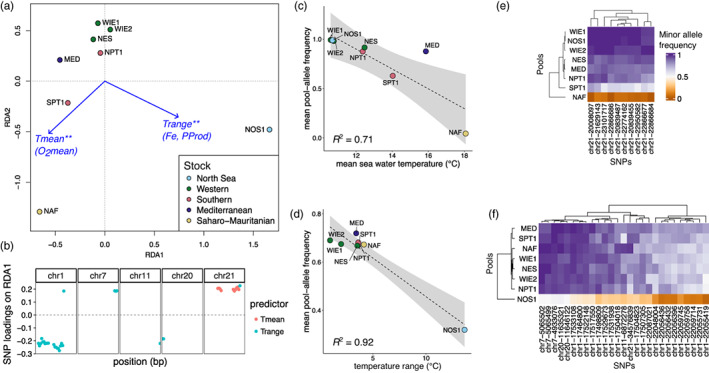
Genome‐by‐environment associations. The adaptively enriched redundancy analysis (RDA) was based on two uncorrelated and statistically significant environmental variables, mean seawater temperature (*T*_mean_) and temperature range (*T*_range_), °C, and the pool‐allele frequencies of the 10 most differentiated SNPs in each of the divergent genomic regions identified with genome scans (*n* = 136). (a) RDA plot. Each point represents a single pool sample, and its color indicates the assigned ICES stock division. The blue arrows represent the loadings of the environmental variables on the first two RDA axes. The statistical significance of environmental variables was tested with 1000 permutations and is indicated with asterisks (**p ≤ 0.01). Highly correlated environmental variables to the ones used in the analysis are shown in parenthesis (Figure S12). (b) Genomic position and loading on the significantly constrained axis RDA1 (*p* ≤ 0.01) of candidate SNPs (with loading >1 SD). Each point represents a single SNP and their color indicates the environmental predictor to which they show the highest correlation. (c, d) A linear relationship between candidate SNPs and the environmental predictor they are most correlated to. (e, f) Heatmap plots depicting the pool‐allele frequencies of candidate SNPs across samples.

The adaptively enriched redundancy analysis (RDA) conducted on 136 putatively adaptive loci identified two main environmental variables strongly associated with genetic differentiation in the Atlantic horse mackerel: mean seawater temperature and temperature range (**p ≤ 0.01, Figure 5a). The first and second significant axes of variation contrasted the North Sea from other localities in the Atlantic Ocean and the western Mediterranean Sea, and the locations north or south of mid‐Portugal following a latitudinal cline. The North Sea is characterized by a higher temperature range as well as higher correlated parameters such as iron content (*R* ^2^ = 0.87) and primary productivity (*R* ^2^ = 0.96, Figure S12). The outlier SNPs that show a strong association with temperature range are located on chr 1, 7, 11, 20, and 22 (Figure 5b,d,f). The latitudinal mean temperature cline is strongly associated with outlier SNPs in the chr 21 inversion (Figure 5b,c,e). Locations north of mid‐Portugal are characterized by colder temperatures (<12°C) than locations in the south (>13°C). Similarly, as seawater temperature has a strong negative correlation with dissolved oxygen (*R* ^2^ = 0.91, Figure S12), the latitudinal gradient is inverse, with higher oxygen content in northern locations and lower oxygen content in southern locations.

### 3.4 Validation of informative markers for genetic stock identification

We found a strong correlation between allele frequencies calculated from individual genotypes and pool‐seq data (mean *R* ^2^ = 0.9 ± 0.1), supporting the findings of the pool‐seq analysis (Figure S13). A total of 72 out of 76 outlier loci, and 157 out of 160 individuals had genotyping success >80% (Table S9). Six SNPs had an indication of deviation from HWE, two markers (12_3119866 and 17_972744) were not polymorphic and one had evident scoring errors where the replicate genotypes in some individuals did not agree (24_5252083), thus these nine markers were excluded. After applying quality filters, the retained data set had 63 SNPs (individual genotypes are shown in Figure S14). Henceforth, this data set will be referred to as the 63‐SNP panel.

To minimize marker redundancy in the 63‐SNP panel, we performed a linkage disequilibrium (LD) analysis for all loci and samples. As expected, significant LD was found between a number of SNPs located in close proximity on the same chromosome (Table S9). Though LD was not statistically significant in some cases (e.g., SNPs in chr 5), these were considered linked due to their physical closeness. To identify the most informative SNPs for sample discrimination while reducing LD, we analyzed *F*_ST_ by marker and by population (Figure S15) for each genomic region. We retained the SNP with the highest average *F*_ST_ per linkage group (assumed to be the most informative), and 155 out of 160 individuals with a genotyping success >80%. The final data set comprised 17 SNPs of which 9 markers are from divergent genomic regions (outlier loci) and 8 markers are neutral (henceforth, the 17‐SNP panel).

An examination of pairwise‐*F*_ST_ values indicated a lack of significant genetic differences between the North Sea samples or between the west of Ireland samples. There was also no significant genetic differentiation between individuals from the west of Ireland, the northern Spanish shelf, and northern Portugal (Table S10).

**FIGURE 6:**
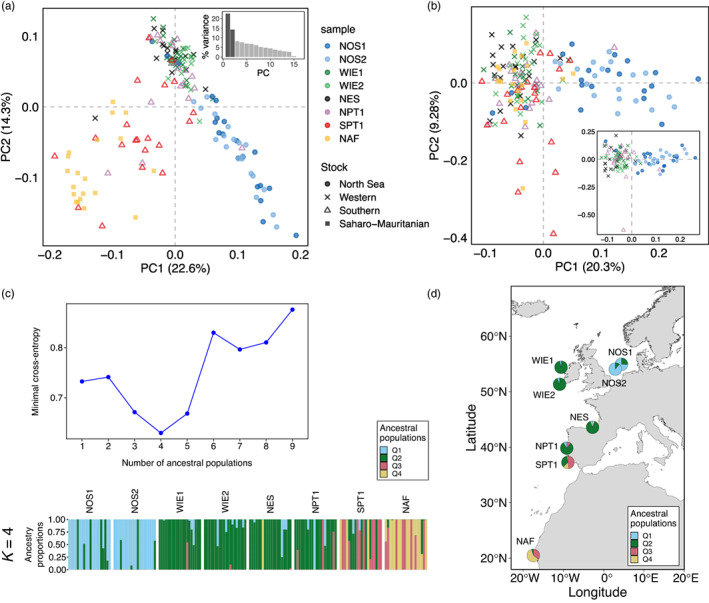
Population structure based on individual genotypes of the 17‐SNP panel. The data set consisted of the genotype of 155 individuals. (a, b) Principal components analysis (PCA) plot based on (a) all 17 markers, (inset) a bar plot showing the percentage (%) of genetic variance explained by each of the first nine principal components (PCs), (b) PCA with all samples but excluding markers from the chr 21 inversion (*n* = 2), (inset) PCA for only the northern samples and excluding markers in the inversion. Only the first two axes are shown per plot. Each dot corresponds to an individual, the dot color indicates the sampling location and the point shape, the designated stock based on the ICES stock divisions. (c) Analysis of admixture, (top) cross‐entropy criterion plot to identify the most likely number of ancestral populations (*K*), (bottom) admixture bar plot for *K* = 4, in which each bar is one individual, and the different colors per bar represent the probability of ancestry for a given *K*. Individuals collected in the same site are grouped as a block, (d) map showing the mean ancestry proportion per location for *K* = 4. Sample names are abbreviated as in Table 1.

The PCA showed that individuals cluster in four main groups: (i) the North Sea; (ii) west of Ireland, northern Spanish shelf, and northern Portugal; (iii) southern Portugal; and (iv) north Africa (Figure 6a). The same groups, but with slightly greater separation, were observed when only using the putatively adaptive SNPs of the panel (in divergent genomic regions, *n* = 9, Figure S16a). When the markers from the chr 21 inversion in the 17‐SNP panel are excluded (*n* = 2), the separation between southern Portugal and north Africa disappears, and the only distinguishable groups are the North Sea and everything else (Figure 6b) or the North Sea and other northern samples (Figure 6b, inset). Therefore, the genotype of the inversion is the main driver of the separation between northern samples, southern Portugal, and North Africa, as it depends on whether individuals are predominantly heterozygous or homozygous for the inversion (Figure S16). While the separation between the four main groups is clear, a few individuals clustered in different groups from the ones expected (Figure 6b, inset).

Individual admixture analysis supports the same four groups identified with PCA (the lowest minimal cross‐entropy value indicates that *K* = 4, Figure 6c, top). In all groups, some individuals showed admixed ancestry, suggesting that they are probably F1‐hybrids or backcrosses between local and migrant individuals. In some cases, the admixed ancestry signal is driven by the haplotype of the chr21 inversion. For example, in southern Portugal, three individuals appear to originate from the western group because they are homozygous for the “northern” haplotype of the inversion, and three individuals seem to originate from the African group because they are homozygous for the “southern” haplotype (Figure 6d, genotypes in Figure S14). Overall, these results indicate that gene flow occurs more often between neighboring geographic areas (Figure 6d).

## 4 DISCUSSION

We generated pooled DNA whole‐genome sequence data (pool‐seq) to examine the population structure, genomic basis, and environmental factors involved in genomic differentiation and local adaptation of the Atlantic horse mackerel. Our results revealed low genome‐wide differences among locations, but high differentiation at a relatively small number of putatively adaptive loci, including a putative chromosomal inversion. The spatial extent of population structure appears to be largely determined by local environmental adaptation rather than spatially constrained gene flow. Although the pool‐seq data results were validated with individual genotyping, the extent of population structure might have been underestimated because about 60% of the collected individuals were not in spawning condition and the pool‐seq method does not allow the identification of genetic heterogeneity within pools. Further studies including larger sample sizes of breeding individuals, and finer geographic coverage are needed to refine the identification of population baselines.

### 4.1 Population structure

We found low but significant genomic differentiation among horse mackerel populations inhabiting the vast geographic area from the North Sea to North Africa (Figure 1a; global mean pool‐*F*_ST_ = 0.007 ± 4.4e‐05). This result is in agreement with previous studies using dozens of neutral genetic markers (Cimmaruta et al., 2008; Comesaña et al., 2008; Healey et al., 2020; Kasapidis & Magoulas, 2008). Low differentiation can be explained by the combined effect of gene flow and large population sizes, implying a minor role of genetic drift in shaping patterns of genetic diversity. In the horse mackerel, gene flow could be mediated by adult migration and by the passive transport of pelagic eggs and larvae by ocean currents over large geographic areas, as the larval stage lasts about one month (Rusell, 1976).

Despite overall low differentiation, we discovered patterns of population structure at the genome‐wide level (Figure 1b–d) that were statistically significant (Figure S9b) and that were supported by loci putatively under selection (Figures 2, 3, 4). Pairwise pool‐*F*_ST_ estimates and PCA revealed three genome‐wide patterns, separating: (i) the western Mediterranean and Atlantic populations, (ii) “northern” and “southern” populations with respect to a genetic break in mid‐Portugal (“northern” samples: North Sea, west of Ireland, northern Spanish Shelf, northern Portugal; “southern” samples: southern Portugal and north of Africa), and (iii) the North Sea respect to other “northern” populations (Figure 1b–d).

Genome scans uncovered a number of genomic regions with elevated differentiation that support these three main subdivisions and further resolve differences among southern samples (Figures 2, 3, 4). Consequently, the five distinguishable genetic groups are (i) the western Mediterranean Sea; (ii) the North Sea; (iii) the west of Ireland, northern Spanish shelf (Bay of Biscay), and northern Portugal; (iv) southern Portugal; and (v) north Africa, Mauritania. These groups appear to be spatially and temporally stable in the short term, as similar allele frequencies were observed at outlier loci in replicate samples collected 1 year apart (west of Ireland WIE1‐WIE2, North Sea NOS1‐NOS2, northern Portugal NPT1‐NPT2, and southern Portugal SPT1‐SPT2; Figures 2, 3, 4). Genomic regions putatively under selection varied in size (70–600 kb, and 9.9 Mb for a putative structural variant) and a complete list of positional candidate genes located within these regions and their putative functions are presented in Table S7.

### 4.2 Genomic evidence separating the western Mediterranean and Atlantic populations

The largest genome‐wide differences were observed between the sample from the western Mediterranean Sea (Alboran Sea) and Atlantic populations (Figures 1b and 2a, mean pool‐*F*_ST_ = 0.011, Figure S9B). This separation was already proposed in earlier studies using body morphometrics, otolith shape, and parasitofauna (Abaunza et al., 2008). However, this is the first genetic evidence supporting the split, as previous studies using microsatellites or mitochondrial DNA were inconclusive (Cimmaruta et al., 2008; Comesaña et al., 2008; Healey et al., 2020; Kasapidis & Magoulas, 2008). The definition of a Mediterranean‐Atlantic genetic divide has been controversial, as it has been reported for some marine species but not for others. A meta‐analysis of 20 phylogeographic studies indicated that such a discrepancy might be due to differences in vicariance and paleoclimate processes and in life‐history traits between species (Patarnello et al., 2007). Likewise, the retentive currents in the Almeria‐Oran front in the western Mediterranean Sea, between Spain and Algeria, have been proposed to act as barriers for gene flow for various marine species (Patarnello et al., 2007). To elucidate which mechanisms are involved in limiting gene flow in this area in the horse mackerel, further sampling within the Mediterranean Sea and collection of individual genomic data are needed.

Interestingly, the pattern of differentiation between the Mediterranean sample and Atlantic populations occurred across the entire genome (Figure 2a), not at particular genomic regions (seen as peaks emerging from the genomic background) as expected when natural selection is the driving force of divergence (e.g., observed in the North Sea and north Africa contrasts, Figures 3 and 4). Such genome‐wide pattern of divergence could be the result of either genetic divergence resulting from limited gene flow followed by genetic drift and/or local adaptation, or the presence of a mixed sample. Previous research based on parasite composition indicated that the Alboran Sea is a mixing area of horse mackerel from the Mediterranean and the Atlantic (Abaunza et al., 2008; Mattiucci et al., 2008). This suggests that the sample of individuals collected in this area could be a mix of Atlantic and Mediterranean individuals.

We detected two outlier loci that distinguish the western Mediterranean Sea, one on chr 5 and the other on chr 21 (Figure 2). The region on chr 21 harbors a single gene, *taar7a* (*trace amine‐associated receptor 7A*; Figure 2b), which encodes a receptor involved in the olfactory sensing of amines (Hashiguchi & Nishida, 2007). The top candidate gene at the chr 5 locus is *opn1mw4* (Figure 2a), a paralog of the *opn1mw* (RH2) gene, which encodes a cone photopigment essential for the vision of blue‐green light. This gene contains two missense mutations (p.Ala284Thr and p.Val224Ile, Figure S17A,B), showing strong genetic differentiation (dAF = 0.53 and dAF = 0.40, respectively). It is possible that the missense mutations in *opn1mw4* may generate a shift in spectral sensitivity similar to the Phe261Tyr substitution in rhodopsin present in many fish species that live in brackish or freshwater (Hill et al., 2019). A change in visual sensitivity could be an adaptive response to the blue‐green light environment in the less turbid waters of the Mediterranean Sea compared to the Atlantic Ocean (Figure S17; Shi & Wang, 2010). Visual adaptation confers survival advantages related to feeding, recognition of conspecifics, and escape from predators.

### 4.3 A putative chromosomal inversion underlies a latitudinal genetic break near mid‐Portugal

Our genomic data revealed a hitherto undescribed genetic break‐off mid‐Portugal, distinguishing populations “northern” or “southern” of this area. This latitudinal pattern was noticeable in pairwise‐*F*_ST_ estimates (Figure 1b, mean pool‐*F*_ST_ = 0.008, Figure S9B), but it was more evident in the PCA based on outlier SNPs (Figure 1d). A large (9.9 Mb) putative inversion on chr 21 underlies the latitudinal genetic pattern (Figure 3). This putative inversion harbors thousands of genes, the roles of which cannot be resolved without further studies. To understand the possible role of the inversion, we examined genome‐environment associations (GEA) with redundancy analysis (RDA). This analysis indicated a strong association between outlier SNPs in the inversion and variation in seawater temperature and/or oxygen content (Figure 5a,b,c,e, Figure S12). Accordingly, the northern haplotype, which is in high frequency among “northern” samples, seems to be associated with colder temperatures (9–12°C) and higher oxygen content (250–266 μmol/m^3^). In contrast, the alternative haplotype dominates in North Africa, where temperatures are the highest (18°C) and oxygen content is the lowest (223 μmol/m^3^). Intermediate haplotype frequencies occur in the south of Portugal, where intermediate temperatures (13–14°C) and oxygen content (242 μmol/m^3^) are common.

The exception to this trend is the prevalence of the northern haplotype in the sample from the western Mediterranean, a location where seawater temperature is higher than expected (16°C vs. 9–12°C among northern samples, Table S8). We cannot verify whether the individuals collected at this location spawn there, as their maturity status at the time of capture is unknown (Table S3). Therefore, it is possible that they may come from a location within the Mediterranean Sea with colder waters comparable to the northern locations. In the Mediterranean Sea, similar temperatures to those in the northern region (~11–12°C) occur in the winter season in coastal waters of the Balearic Sea near Catalonia (Spain), the Gulf of Lyon (France), and the Ligurian Sea (northeast Italy; Pastor et al., 2020). Indeed, previous studies have reported spawning events of horse mackerel near Catalonia during the winter months (Andreu & Rodriguez‐Roda, 1953; Planas & Vives, 1953).

The GEA results are consistent with the observation that the inferred location of the latitudinal genetic break coincides with a major biogeographical transition zone between temperate and subtropical waters off the coast of central Portugal, near Lisbon (~38.7–39.0°N; Cunha, 2001; Santos et al., 2007). A genetic study in the boarfish (*Capros aper*), a pelagic fish with similar distribution and life history characteristics as the horse mackerel, reported a comparable latitudinal pattern (Farrell et al., 2016). Thus, it is possible that the environmental transition zone in mid‐Portugal is a major driver of population structuring for several marine species inhabiting this area, including the horse mackerel.

### 4.4 A distinct population in the southern North Sea

Our genomic data demonstrated that there is a genetically distinct population in the southern North Sea (Figure 1b,d, mean pool‐*F*_ST_ = 0.011, Figure S9b). This adds to previous morphometric and parasite data suggesting that horse mackerel from this area differs from nearby Atlantic populations (Abaunza et al., 2008).

Genome scans revealed seven genomic regions that distinguish the North Sea (Figure 4a). The replicate samples from this area showed similar genome‐wide backgrounds (pool‐*F*_ST_ = 0.001, Figure S9b) and nearly identical allele frequencies at outlier loci (Figure 4b–e). However, these replicate samples likely represented the same cohort, as indicated by their length–frequency and maturity stages (Table S3), meaning that they were not independent observations but support the presence of short‐term stability of the horse mackerel in this area.

Some of the positional candidate genes for local adaptation to the North Sea are *gpr83*, *sgms2*, *ncoa2*, and *taar7a* (Figure 4). *gpr83* (G‐protein coupled receptor 83) encodes a receptor that plays a role in the regulation of energy metabolism, feeding, reward pathway, and stress/anxiety responses in mice (Gomes et al., 2016; Lueptow et al., 2018). *ncoa2* (*nuclear receptor coactivator 2*) encodes a transcriptional coactivator for steroid receptors that are presumably involved in glucose metabolism regulation (Bateman et al., 2021). Previous experimental studies indicate that fish adapted to cold climates often have higher metabolic rates than those adapted to warm climates (Wang et al., 2014; White et al., 2012). Thus, selection may favor alleles that result in increased energy metabolism required for adaptation to the cold environment of the North Sea. *sgms2* (*sphingomyelin synthase 2*) on chromosome 1 encodes a protein involved in the synthesis of sphingomyelin, a major component of cell and Golgi apparatus membrane. Previous studies indicate that this protein is crucial to maintain cell membrane structure and fluidity at low temperatures in fish (Wang et al., 2014; Windisch et al., 2011). *taar7a* (*trace amine‐associated receptor 7A*) encodes an olfactory receptor specific for sensing amines in vertebrates (Hashiguchi & Nishida, 2007; Hussain et al., 2009; Tessarolo et al., 2014; Yamamoto et al., 2010). Interestingly, the North Sea and the Mediterranean samples tended to be fixed for alternate haplotypes at this locus (Figure 4d). Amines are odorants proposed to play a critical role in intra‐ and inter‐specific communication in, for example, sexual attraction or avoidance of predators or rotting food (Dewan, 2021). A study on two goatfish species with contrasting bottom habitat preferences (*Mullus surmuletus* and *Mullus barbatus*) reported significant differences in the morphology of chemoreceptors (Lombarte & Aguirre, 1997). Such differences are proposed to be associated with increased sensitivity to chemical stimuli in species living in muddy deeper waters, where visual capabilities are reduced. Among the locations in the east Atlantic included in this study, the North Sea has the highest water turbidity of all (Figure S17, Shi & Wang, 2010). Thus, it is possible that natural selection may favor *taar7a* alleles that confer an enhanced sense of smell under the reduced visibility in the North Sea.

The GEA analysis indicated that there is a strong association between outlier SNP characteristics of the North Sea and variation in temperature range or correlated environmental parameters such as iron content and primary productivity (Figure 5a,b,d,f, Figure S12). The North Sea corresponds to the northern limit of the reproductive range of the species and exhibits a combination of environmental factors that makes this area unique. The North Sea is characterized by colder mean temperatures and a higher temperature range (colder winters and warmer summers) than other locations included in this study as well as higher oxygen content, iron content, and primary productivity. The particular environmental conditions in this area and the number of genomic regions that appear to be under selection suggest a polygenic response to diverse selection pressures driving local adaptation.

### 4.5 Evolutionary implications

A long‐standing question in evolutionary biology and conservation is what is the spatial scale at which population subdivision occurs in highly mobile marine species. Based on this study and previous research, we propose that population structuring in marine species could be largely determined by the strength and selective pressures imposed by environmental factors experienced at crucial life stages that determine survival and fitness.

We reached this conclusion by comparing the life history and population structure patterns of Atlantic horse mackerel, Atlantic herring, and European eel, three migratory marine species analyzed with whole‐genome sequencing. The number of loci involved in ecological adaptation in the Atlantic horse mackerel and their degree of genetic differentiation is small compared with those in the Atlantic herring (Han et al., 2020) but is intermediate to the Atlantic herring and the European eel, as the latter constitutes a single panmictic population (Enbody et al., 2021). We propose that the most important explanation for the differences in genetic structuring between these three species is related to their respective spawning strategies because spawning and early development constitute the most sensitive period of life for a fish, characterized by high mortality (Dahlke et al., 2020) and thus strong selection. The Atlantic herring is a demersal spawner that breeds close to the coast in areas with marked environmental differences between populations as regards temperature, salinity, depth, and biotic conditions (plankton production, predators, etc.). It is also presumed to show prevalent homing behavior, that is, individuals return for spawning to the locations where they were hatched. In contrast, the Atlantic horse mackerel is a benthopelagic fish that spawns in deeper waters, near the shelf edge (100–200 m deep), at not well‐defined spawning areas. This implies that the environmental conditions in which breeding occurs are comparatively less diverse. On the other extreme, to the best of our knowledge, all European eels spawn in the Sargasso Sea under similar environmental conditions.

### 4.6 Implications for fisheries assessment and management

The genetic‐based groups identified here are largely in agreement with the current horse mackerel stocks, informed by the results of the 2000–2003 HOMSIR project (Abaunza et al., 2008). However, our data do not support the current definition of the Southern stock in Portuguese waters (Figure S1) and of the southern boundary of the Western stock. Our genomic data indicate that the Southern stock might not have well‐defined boundaries but rather constitutes a contact zone between at least two diverse biological units (Figures 1, 3 and 6). Samples from northern Portugal (north of Lisbon, ~38.7–39.0 °N) appear to be genetically closer to the Western stock, while samples from southern Portugal (south of Lisbon) form their own group but are genetically closer to the samples from the Saharo‐Mauritanian stock, in North Africa. To confirm these findings and assess the spatial and temporal trends of mixing between these areas, further studies are required, including a finer geographic sampling and screening of informative genetic variants in a large number of individuals throughout this area. We did not find significant genetic differences between northern Portugal (currently considered part of the Southern stock), northern Spanish shelf (Bay of Biscay), and the west of Ireland, implying that the southern boundary of the Western stock could possibly be extended down to northern Portuguese waters. However, the minute genetic differentiation does not exclude the possibility that isolation in an ecologically relevant timescale of interest for fisheries management might occur (Hauser & Carvalho, 2008).

Finally, our genomic data support the consideration of the Mediterranean Sea as a separate stock, as proposed by the HOMSIR project (Abaunza et al., 2008). While a single sample from the westernmost part of the Mediterranean was studied, its genetic distinctiveness suffices to infer that the Mediterranean horse mackerel likely constitutes a separate population from those in the Atlantic. Wide‐scale sampling within the Mediterranean Sea is required to further explore the population structure in this region.

This study identified a number of genetic markers (SNPs) that can be used as a genetic tool for fisheries stock assessment. A panel of only 63 markers suffices to identify the main genetic subdivisions. In fact, using a reduced panel of only 17 markers, it is possible to differentiate individuals collected in the North Sea and North Africa from neighboring populations. These markers can help, for instance, to elucidate the extent of mixing between the Western and North Sea stocks in the English Channel (ICES Divisions 7.e and 7.d) and in ICES area 4.a in the northern North Sea. Therefore, this study can serve as a successful example of the utility of genetic tools for fisheries monitoring and management.

## CONFLICT OF INTEREST STATEMENT

The authors declare no competing interest.

## Supporting information


Data S1

## Data Availability

Sequence data generated in this study are available in the NCBI Short Read Archive (SRA) under BioProject PRJNA957442. Pool‐wise allele frequencies, environmental data, and individual genotypes are available at the SciLifeLab Data repository DOI: 10.17044/scilifelab.22670290. Custom scripts are available in the Github repository: https://github.com/LeifAnderssonLab/2023_Horse_mackerel_popgen.
